# *In silicio* expression analysis of *PKS* genes isolated from *Cannabis sativa* L.

**DOI:** 10.1590/S1415-47572010005000088

**Published:** 2010-12-01

**Authors:** Isvett J. Flores-Sanchez, Huub J. M. Linthorst, Robert Verpoorte

**Affiliations:** 1 Gorlaeus Laboratories, Pharmacognosy Department/Metabolomics, Institute of Biology Leiden, Leiden University, LeidenThe Netherlands; 2 Clusius Laboratory, Institute of Biology Leiden, Leiden University, LeidenThe Netherlands

**Keywords:** *Cannabis sativa*, homology modeling, polyketide synthases, RT-PCR

## Abstract

Cannabinoids, flavonoids, and stilbenoids have been identified in the annual dioecious plant *Cannabis sativa* L. Of these, the cannabinoids are the best known group of this plant's natural products. Polyketide synthases (PKSs) are responsible for the biosynthesis of diverse secondary metabolites, including flavonoids and stilbenoids. Biosynthetically, the cannabinoids are polyketide substituted with terpenoid moiety. Using an RT-PCR homology search, PKS cDNAs were isolated from cannabis plants. The deduced amino acid sequences showed 51%-73% identity to other CHS/STS type sequences of the PKS family. Further, phylogenetic analysis revealed that these PKS cDNAs grouped with other non-chalcone-producing PKSs. Homology modeling analysis of these cannabis PKSs predicts a 3D overall fold, similar to alfalfa CHS2, with small steric differences on the residues that shape the active site of the cannabis PKSs.

## Introduction

In plants, polyketide synthases (PKSs) play an important role in the biosynthesis of a myriad of secondary metabolites (Schröder, 1997; Flores-Sanchez and Verpoorte, 2009). PKSs are a group of homodimeric condensing enzymes that catalyze the initial key reactions in the biosynthesis of several compounds, such as flavonoids and stilbenoids. PKSs are classified into three types (Hopwood and Sherman, 1990; Staunton and Weissman, 2001; Fischbach and Walsh, 2006). Chalcone synthase (CHS, EC 2.3.1.74) and stilbene synthase (STS, EC 2.3.1.95) are the most studied enzymes from the group of type III PKSs (Schröder, 2000; Austin and Noel, 2003). Plant PKSs have 44%-95% amino acid identity and are encoded by similarly structured genes. For example, CHSs from *Petunia hybrida*, *Petroselinum hortense*, *Zea mays*, *Antirrhinum majus*, and *Hodeum vulgare*, and STS from *Arachis hypogaea* have 70%-75% identity on the protein level and the *CHS* and *STS* genes contain an intron at the same conserved position (Schröder *et al.*, 1988; Schröder and Schröder, 1990). Families of PKS genes have been reported in many plants, such as *Daucus carota* L. (Hirner and Seitz, 2000), *Gerbera hydrida* (Helariutta *et al.*, 1996), *Glycine max* (Shimizu *et al.*, 1999), *Humulus lupulus* (Novak *et al.*, 2006), *Hypericum androsaemum* (Liu *et al.*, 2003), *Ipomoea purpurea* (Durbin *et al.*, 2000), *Lycopersicon esculentum* (O'Neill *et al.*, 1990), *Medicago sativa* L. (Junghans *et al.*, 1993), *Petunia hybrida* (Koes *et al.*, 1989), *Phaseolus vulgaris* (Ryder *et al.*, 1987), *Pinus sylvestris* L. (Preisig-Muller *et al.*, 1999), *Pisum sativum* (Harker *et al.*, 1990), *Psilotum nudum* (Yamazaki *et al.*, 2001), *Rheum palmatum* (Abe *et al.*, 2005), *Rubus idaeus* (Kumar and Ellis, 2003), *Ruta graveolens* (Springob *et al.*, 2000), *Saccharum spp.* (Contessotto *et al.*, 2001), *Sorghum bicolor* (Lo *et al.*, 2002), *Vitis vinifera* (Wiese *et al.*., 1994; Goto-Yamamoto *et al.*, 2002), and *Zingiber officinale* (Radhakrishnan and Soniya, 2009). Their expression is controlled differently and it has been suggested that PKSs have evolved by gene duplication and, subsequently, divergence by mutations, providing an adaptative differentiation to plants (Tropf *et al.*, 1994; Durbin *et al.*, 2000; Lukacin *et al.*, 2001). As PKSs are in vital branch points for the biosynthesis of secondary metabolites, the presence of families of PKSs in one single species emphasizes the importance of their characterization to understand their functional divergence and their contribution to function(s) in different cell types of the plant.

*Cannabis sativa* L. is an annual dioecious plant from Central Asia. Several compounds have been identified in this plant. Cannabinoids are the best known group of natural products and more than 70 different cannabinoids have been found so far (ElSohly and Slade, 2005; Radwan *et al.*, 2008). Several therapeutic effects of cannabinoids have been described (Williamson and Evans, 2000) and the discovery of an endocannabinoid system in mammals marked a renewed interest in these compounds (Mackie, 2008; Moreira and Lutz, 2008; Heifets and Castillo, 2009). However, other groups of secondary metabolites have also been described, such as flavonoids and stilbenoids (Flores-Sanchez and Verpoorte, 2008b). It is known that the PKSs CHS and STS catalyze the first committed step of the flavonoid and stilbenoid biosynthesis, respectively. Cannabinoid biosynthesis can be initiated by a PKS (Shoyama *et al.*, 1975). Previously, a PKS cDNA was generated from *C. sativa* leaves. It encodes an enzyme with CHS, phlorisovalerophenone synthase (VPS), and isobutyrophenone synthase (BUS) activities, but lacking olivetolic acid synthase activity (Raharjo *et al.*, 2004). The co-existence of cannabinoids, flavonoids, and stilbenoids in *C. sativa* could be correlated to different enzymes of the PKS family. Analyses of crude protein extracts from cannabis plant tissues have revealed the presence of PKS enzymatic activities. Multiple PKS activities were detected during the development and growth of glandular trichomes on bracts and the content analyses of cannabinoids and flavonoids revealed differences in their distribution in these glandular tissues (Flores-Sanchez and Verpoorte, 2008a). This report deals with the generation and molecular analyses of PKS cDNAs obtained from messenger RNA from glandular tissues of cannabis plants in order to obtain a PKS gene library for future studies. Homology modeling, motif, and phylogenetic analyses were used for an *in silicio* expression analysis.

## Material and Methods

###  Plant material

Seeds of *Cannabis sativa*, Skunk and Fourway drug-type varieties (The Sensi Seed Bank, Amsterdam, the Netherlands), and Kompolti fiber-type variety (Dr. D. Watson, HortaPharm, Amsterdam, the Netherlands) were germinated and 9-day-old seedlings were planted into 11 LC pots with soil (substrate 45 L, Holland Potgrond, Van der Knaap Group, Kwintsheul, the Netherlands) and maintained under a light intensity of 1930 lux, at 26 °C and a 60.02 ± 7.43% relative humidity (RH). After three weeks the small plants were transplanted into 10 L pots for continued growth until flowering. To initiate the flowering, two-month-old plants were transferred to a photoperiod chamber (12 h light, 27 °C, and 37.0 ± 11.6% RH). 3-month-old male plants were used to pollinate female plants. 5-day-old seedlings, young leaves from 13-week-old plants, female flowers in different stages of development, fruits from 18-day-old plants and male flowers from 4-month-old plants were harvested. Roots from 4-month-old female plants were harvested and washed with cold water to remove residual soil. In addition, cones of *H. lupulus* at different stages of development were collected in September 2004 from the Pharmacognosy gardens (Leiden University). All vegetal material was weighed and stored at -80 °C.

###  Isolation of glandular hairs and lupulin glands

Six grams of frozen female flowers containing 17-, 23-, 35-, and 47-day-old glandular trichomes from cannabis plants were removed by shaking frozen material through a tea leaf sieve and collecting it in a mortar containing liquid N_2_, where it was immediately used for RNA extraction. The effectiveness of this method is comparable to the method reported by Yerger *et al.* (1992), which consists of shaking the tissues with powdered dry ice and sieving. For lupulin glands, frozen cones of hop were ground in liquid nitrogen using a frozen mortar and pestle only to long enough to separate the bracteoles and then were shaken using the same system as for cannabis glandular hairs.

###  Total RNA and mRNA isolation

For total RNA isolation from flowers, leaves, roots, seedlings, fruits, glandular hairs, glandular lupulins, and hop cones, frozen tissues (0.1-0.5 g) were ground to a fine powder in a liquid-nitrogen-cooled mortar, suspended and vortexed in 0.5 mL of extraction buffer (0.35 M glycine, 0.048 M NaOH, 0.34 M NaCl, 0.04 M EDTA, and 4% SDS) and 0.5 mL of water-saturated phenol. The suspension was centrifuged at 1,400 rpm for 2 min to separate the phenol and water phases. The RNA was precipitated in 1/3 volume 8 M LiCl at 4 °C overnight. The RNA was collected by centrifugation at 14,000 rpm for 10 min and resuspended in 0.1 mL of H_2_O. The suspension was heated at 60 °C for 20 min and centrifuged. A total of 5 μL of 3 M Na-acetate (pH 4.88) was added to the supernatant to initiate the precipitation with 0.25 mL of 100% EtOH at -20 °C for 30 min and centrifuged at 14,000 rpm for 7 min. The pellet was washed with 250 μL of 70% EtOH, centrifuged for 2 min at 14,000 rpm, dried at 60 °C for 15 min, dissolved in 50 μL of H_2_O, and incubated at 50 °C for 10 min.

Alternatively, Micro-Fast Track 2.0 kit and Trizol Reagent (Invitrogen, Carlsband, CA, USA) were also used for mRNA and total RNA isolation following the manufacturer's instructions. Isolated RNA was stored at -80 °C.

###  RT-PCR

Degenerated primers, HubF (5'-GAGTGGGGYCA RCCCAART-3'), HubR (5'-CCACCIGGRTGWGYAAT CCA-3'), CHSF (5'-GGYTGYIIIGSYRGTGGMA-3'), CHSR (5'-CCIGGYCCRAASCCRAA-3'), STSF (5'-GGITGCIIIGCIGGIGGMAC-3'), and STSR (5'-CCIGGI CCRAAICCRAA-3') (Biolegio BV, Malden, the Netherlands) were designed based on CHS, STS, and stilbene carboxylate synthase (STCS) sequences from *H. lupulus*, *A. hypogaea*, *Rheum tataricum*, *Pinus strobus*, *V. vinifera* and *H. macrophylla*. For primers HubF, HubR, CHSF, and CHSR, the conserved regions were from CHS and VPS (GenBank accession nos. AJ304877, AB061021, AB061022, AJ430353, and AB047593), while for STSF and STSR they were from STS and STCS (GenBank accession nos. AB027606, AF508150, Z46915, AY059639, AF456446). RT-PCR was performed with total RNA or mRNA as a template using different combinations of primers. Reverse transcription was performed at 50 °C for 1 h followed by deactivation of the ThermoScript Reverse Transcriptase (Invitrogen) at 85 °C for 5 min. The PCR conditions were: 5 cycles of denaturation for 45 s at 94 °C, 1 min of annealing at 40 °C, 1 min of DNA synthesis at 72 °C, followed by 5 cycles with annealing at 41 °C and 5 cycles with annealing at 43 °C, and ending with 35 cycles with annealing at 45 °C. A Perkin Elmer DNA Thermal Cycler 480 and a Taq PCR Core kit (QIAGEN, Hilden, Germany) were used. A final extension step for 10 min at 72 °C was included. The PCR products were separated on 1.5% agarose gel, visualized under UV light, and recovered using Zymoclean Gel DNA Recovery kit (Zymo Research, Orange, CA, USA) or QIAquick PCR Purification kit (QIAGEN) according to the manufacturer's instructions.

###  RACE-PCR

For generation of 5' and 3' end cDNAs, we used total RNA, gene specific primers, and a SMART RACE kit (ClonTech, Palo Alto, CA, USA). The cycling parameters were: 94 °C for 1 min followed by 35 cycles at 94 °C for 35 s, annealing temperature for 35 s and 72 °C for 3 min. A final elongation step for 10 min at 72 °C was included. Gene-specific, amplification, and sequencing primers and annealing temperatures are shown in Table S1 (Supplementary Material). The PCR products were separated on 1.5% agarose gel and visualized under UV light. For generation of complete sequences, total RNA and amplification primers were used. Nested amplifications were made with gene-specific primers to select PKS sequences for sequencing. PKS full-length cDNAs were re-sequenced with sequencing primer in order to confirm that the ORFs of the sequences were correct. The corresponding amplification products were ligated into pGEM-T vector and cloned into JM109 cells according to the manufacturer's instructions (Promega, Madison WI, USA). Plasmids containing the inserted fragment were sequenced (BaseClear, Leiden, the Netherlands).

###  Homology modeling

The PKS 3D models were generated by the web server Geno3D (Combet *et al.*, 2002), using as template the X-ray crystal structures of *M. sativa* CHS2 (Protein Bank accession nos. 1BI5.pdb, 1CHW.pdb, and 1CMl.pdb). The models were based on the sequence homology of residues Arg5-Ile383 of the PKSs PKSG1, PKSG2, PKSG4, PKSG5, and PKSF3. The VPS model was based on the sequence homology of the residues Val4-Val390. The corresponding Ramachandran plots confirm that the majority of residues grouped in the energetically allowed regions. All models were displayed and analyzed by the program DeepView - Swiss-PdbViewer (Guex and Peitsch, 1997).

## Results and Discussion

###  Amplification of cannabis *PKS* cDNAs

RNA isolated from glandular hairs of cannabis flowers and plant tissues was used as a template for reverse transcription-polymerase chain reaction (RT-PCR) amplification of segments of *PKS* mRNAs. RNA from hop tissues was used as a positive control. The degenerated primers corresponded to conserved regions surrounding Gln 119, the catalytic domain around Cys 164, a region surrounding His 303, and the *C*-terminal region of the selected protein sequences from CHS, STS, and STCS. Amplification with different primer combinations yielded products of expected size (Figure S1, Supplementary Material). Nucleotide sequence analysis showed open reading frames (ORFs) encoding for PKS proteins. Amplifications derived from mRNA of leaves, seedlings, glandular hairs, and female and male flowers showed 76%-78% homology with CHS3 from *H. lupulus* and 66%-69% homology with the known cannabis CHS-type PKS (Raharjo *et al.*, 2004). This CHS-type PKS was also identified in female and male flowers and in glandular hairs. Partial sequences of VPS and CHS2 from the hop cone's secretory glands (also called lupulin glands) were obtained. It is known that *VPS* and *CHS1* are expressed in lupulin glands (Okada and Ito, 2001; Matousek *et al.*, 2002a,b) and the presence of a gene family of *VPS,* as well as one of *CHS,* has been suggested. Supplementary Figure S2 shows the strategy to obtain the full-length cDNAs of the likely PKS gene(s) differing from the earlier CHS-type PKS gene.

###  Nucleotide and protein sequence analyses

Several full-length *PKS* cDNAs containing ORFs of 1158 bp were obtained (Table 1). The main difference among these *PKS* cDNAs was the size of untranslated regions (UTRs). Several studies suggest that the untranslated regions (UTRs) are important for the control of gene expression in plants at the post-transcriptional level. Stability (Feldbrugge *et al.*, 2001; Schwartz *et al.*, 2006; Vaucheret, 2006), transport (Li and Hunt, 1997; Siomi, 2000), and translation (Klaff *et al.*, 1996; Guo *et al.*, 2000; Geslain and Ribas de Pouplana, 2004) of RNA depend on the 5'-UTR, the 3'-UTR, or both. We believe that variation in the size of UTRs from these *PKS* cDNAs could be the result of alternative transcription initiation and polyadenylation sites from post-transcriptional processing of *PKS* pre-mRNAs (Dean *et al.*, 1986; Joshi, 1987; Rothnie, 1996). The nucleotide sequence data were deposited at GenBank database with the GenBank accession numbers EU551163 (PKSG1), EU551164 (PKSG2), EU551162 (PKSF3), EU551165 (PKSG4), and EU551166 (PKSG5).

**Figure 1:**
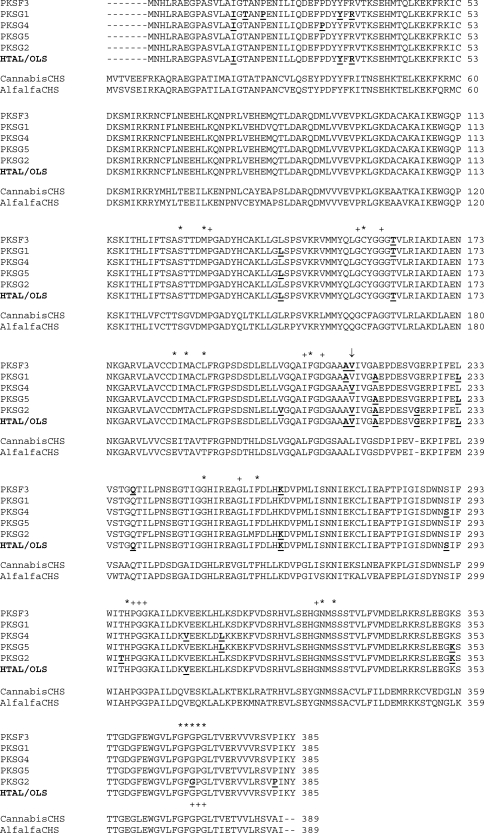
Comparison of the deduced amino acid sequences of *C. sativa* PKSs and *M. sativa* CHS2. Amino acid residues from catalytic triad (Cys164, His303, and Asn 336), starter substrate-binding pocket (Ser133, Glu192, Thre194, Thre197, and Ser338), “gatekeepers” (Phe215 and Phe265), and others important for functional diversity (GFGPG loop, Gly256, and Met137) are marked with *. Residues that shape the geometry of the active site are marked with +. Amino acids in bold and underlined have different codon; differences on amino acid sequence are highlighted in gray; ↓, three different codons for Val (numbering in *M. sativa* CHS2).

The ORFs encode proteins of 385 amino acids with a calculated MW of approximately 42 kDa and a pI ranging from 5.98 to 6.09 (Table 2). The predicted amino acid sequences have more than 97% homology (Figure 1). In the plant *H. lupulus*, three *CHS 1* mRNA sequences were isolated that shared more than 99% and 98% identity on nucleotide and amino acid levels, respectively. They are clearly homologous to the original *CHS 1* sequence (AJ304877), forming a *CHS 1* oligofamily (Matousek *et al.*, 2006). The presence of this oligofamily could promote differences in concentration of prenylflavonoids in different varieties of *H. lupulus* (De Keukeleire *et al.*, 2003; Matousek *et al.*, 2005, 2010).

According to the percentage of identity at amino acid level (Table S2), our five PKSs showed to have more homology with the CHSs 3, 4, and VPS from *H. lupulus* than other PKSs (70%-73%). Conserved amino acid residues present in type III PKSs are also preserved in the amino acid sequences from our PKSs (Figure 1). The catalytic triad (Cys157, His297, and Ans330), the “gatekeeper” phenylalanines (Phe208 and Phe259), and Met130, which ties one catalytic site to the other one on the homodimeric complex, as well as Gly250, which determines the elongation cavity volume of the active site, are strictly preserved when compared to CHS2 from alfalfa (Ferrer *et al.*, 1999; Jez *et al.*, 2000a; Jez *et al.*, 2001). The GFGPG loop, which is important for the cyclization reactions in CHS/STS-type PKSs (Suh *et al.*, 2000), is also preserved on our PKSs. In the starter substrate-binding pocket, the amino acid residues Ser126 and Ser332 are also preserved as on alfalfa CHS2, but Glu185, Thr187 and Thr190 are replaced by an Asp, a Met and a Leu, respectively. Only in PKSG2 is the residue Thr187 preserved as on alfalfa CHS2. In the PKS 2-pyrone synthase (2PS), the amino acid residue Thr190 is replaced by a Leu. All these amino acid residues are important for the selectivity of the starter substrate. In alfalfa CHS2, the catalytic efficiency of the *p*-coumaroyl-CoA-binding pocket was affected by replacement of these residues (Jez *et al.*, 2000b). The replacement of Thr197 by Leu slightly reduced its catalytic efficiency for the substrate *p*-coumaroyl-CoA. However, it was increased for the substrate acetyl-CoA. It was found that the change of three amino acid residues (Thr197Leu, Gly256Leu, and Ser338Ile) converts a CHS activity to 2PS activity. In our PKSs, the substrate-binding pocket could be slightly different from that of the alfalfa CHS2 by changes from polar to nonpolar amino acid residues (Thr187Met and Thr190Leu) and from length and bulkiness of the side chain residues (Glu185Asp185). Although the residues that shape the geometry of the active site (Pro131, Gly156, Gly160, Asp210, Gly256, Pro298, Gly299, Gly300, Gly329, Gly368, Pro369, and Gly370) are preserved on alfalfa CHS2 (Ferrer *et al.*, 1999), Leu209 is replaced by the amino acid Ile.

**Figure 2:**
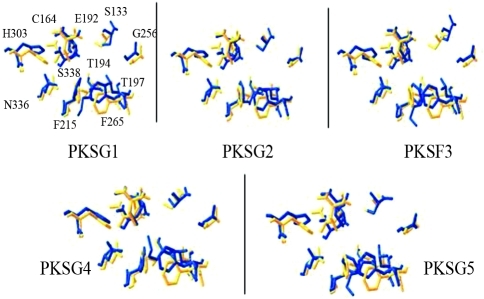
The relative orientation of the side chains of the active site residues from *M. sativa* CHS with the 3D models of *C. sativa* PKS. The corresponding side chains in alfalfa CHS are shown in yellow backbones and are numbered.

The CHS-based homology modeling predicted that our cannabis PKSs would have the same three-dimensional overall fold as alfalfa CHS2 (Figure S3). A schematic representation of the residues that shape the geometry of the active site of cannabis PKSs is shown in Figure 2. The models suggest small differences in the local reorientation of the residues that shape the active site of the cannabis PKSs. The substrate and product specificity of the enzyme reaction can be affected by the steric modulation of the active-site architecture (Ferrer *et al.*, 1999; Jez *et al.*, 2000a,b, 2001; Suh *et al.*, 2000). It can be inferred from these models that cannabis PKSs could have differences in substrate specificity or in catalytic efficiency (*k*

_cat_/*K*_*M*_). In PKF3 and PKSG5 the Phe208 and Phe259, which are situated at the active site entrance, seem to be closer together than those in the other PKSs. In PKSG1, PKSG2, and PKSG4, the active site entrance looks narrower than that at CHS2. Jez *et al.* (2002) reported that the replacement F265V increased the preference for aliphatic CoA starters in CHS2, but the replacement F215S yielded a CHS mutant that accepts *N*-methylanthraniloyl-CoA as a substrate. In acridone synthase (ACS) from *Ruta graveolens,* the exchange of Val265Phe reduced the catalytic activity and shifted the starter substrate preference (*N*-methylanthraniloyl-CoA) to *p*-coumaroyl-CoA, whereas a triple replacement from the residues Ser132Thr, Ala133Ser, and Val265Phe transformed the ACS to a functional CHS (Lukacin *et al.*, 2001, 2005). On the other hand, it has been suggested that Phe215 may help orient substrates at the active site during elongation of the polyketide intermediate and that the position of the CoA's terminal thiol may affect the conformations of Phe215 and Phe265 (Jez *et al.*, 2000a). A PKS isolated from *Polygonum cuspidatum* showed a pH-dependent activity and its gatekeepers, Phe215 and Phe265, are replaced by Leu and Cys, respectively. It showed a preference for aromatic CoA esters and could not accept isobutyryl-CoA, isovaleryl-CoA, or acetyl-CoA as substrates (Ma *et al.*, 2009). The residues Leu214 and Phe215 are replaced by Ile214 and Leu215 in benzalacetone synthase (BAS). These residues are involved in the formation of benzalacetone in *R. palmatum* (Abe *et al.*, 2003). On the other hand, Thr197, Gly256, and Ser338, which are replaced by Ala, Leu, and Thr, respectively, in aloesone synthase (ALS) have different roles in the formation of the heptakedite aloesone in *R. palmatum.* Gly256 determines starter substrate selectivity, Thr197 controls polyketide chain length, and Ser338 guides the linear polyketide intermediate into the pocket and leads the formation of aloesone (Abe *et al.*, 2006). In cannabis PKSs, the residues Ser126, Leu190, Gly250, and Ser332 have changes in their orientation (Figure 2). As was mentioned above, these minor changes could have drastic effects on the enzymatic activity of each cannabis PKS.

Motif analyses predicted PKSG1, 2, 4, 5 and PKSF3 to be non-secretory proteins with a putative cytoplasmic location. In addition, potential residues for post-translational modifications, such as phosphorylation and glycosylation, were also predicted. Biochemical analyses are required to prove that these PKSs have a cytoplasmic localization and can be modified by glycosylation and/or phosphorylation.

###  A PKS family in cannabis plants

**Figure 3:**
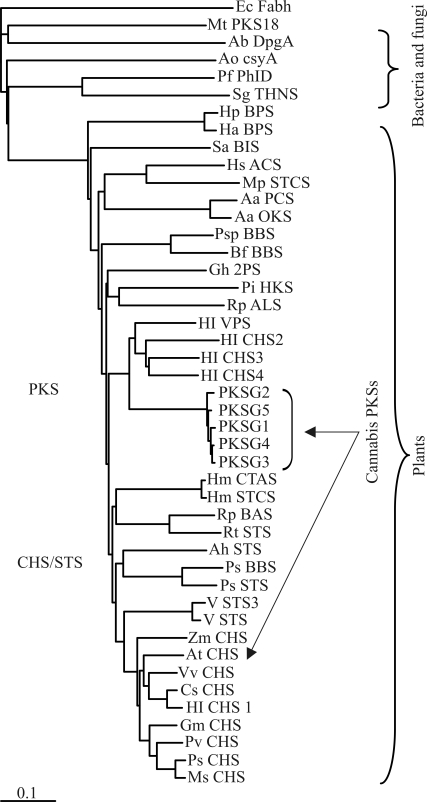
Relationship of *C. sativa* PKSs to plant, fungal, and bacterial type III PKSs. The tree was constructed with III type PKS protein sequences. *E. coli* β-ketoacyl synthase III (Ec_Fabh, ProteinBank accession no. 1EBL) was used as out-group. Multiple sequence alignment was performed with CLUSTALW (1.83) program and the tree was displayed with TreeView (1.6.6) program. The indicated scale represents 0.1 amino acid substitution per site. Abbreviations: Mt_PKS18, *Mycobacterium tuberculosis* PKS18 (AAK45681); Ab_DpgA, *Amycolatopsis balhimycina* DpgA (CAC48378); Ao_csyA, *Aspergillus oryzae* csyA (BAD97390); Pf_PhlD, *Pseudomonas fluorescens* phlD (AAB48106); Sg_THNS, *Streptomyces griseus* (BAA33495); Hp_BPS, *Hypericum perforatum* BPS (ABP49616); Ha_BPS, *Hypericum androsaeum* BPS (AAL79808); Sa_BIS, *Sorbus aucuparia* BIS (ABB89212); Hs_ACS, *Huperzia serrata* ACS (ABI94386); Mp_STCS, *Marchantia polymorpha* STCS (AAW30010); Aa_PCS, *Aloe arborescens* PCS (AAX35541); Aa_OKS, *A. arborescens* (AAT48709); Psp_BBS, *Phalaenopsis* sp. ‘pSPORT1' BBS (CAA56276); Bf_BBS, *Bromheadia finlaysoniana* BBS (CAA10514); Gh_2PS, *Gerbera hybrida* 2PS (P48391); Pi_HKS, *Plumbago indica* HKS (BAF44539); Rp_ALS, *Rheum palmatum* ALS (AAS87170); Hl_VPS, *Humulus lupulus* VPS (BAA29039); Hl_CHS2, *H. lupulus* CHS2 (BAB47195); Hl_CHS3, *H. lupulus* CHS3 (BAB47196); Hl_CHS4, *H. lupulus* CHS4 (CAD23044); Hm_CTAS, *Hydrangea macrophylla* CTAS (BAA32733); Hm_STCS, *H. macrophylla* STCS (AAN76182); Rp_BAS, *R. palmatum* BAS (AAK82824); Rt_STS, *Rheum tataricum* STS (AAP13782); Ah_STS, *Arachis hypogaea* STS (BAA78617); Ps_BBS, Pinus sylvestris BBS (pinosilvin synthase, CAA43165); Ps_STS, *Pinus strobus* STS (CAA87013); V_STS3, *Vitis* sp. cv. ‘Norton' STS3 (AAL23576); V_STS, *Vitis* spp. STS (AAB19887); Zm_CHS, *Zea mays* CHS (AAW56964); Gm_CHS, *Glycine max* CHS (CAA37909); Pv_CHS, *Phaseolus vulgaris* CHS (CAA29700); Ps_CHS, *Pisum sativum* CHS (CAA44933); Ms_CHS, *Medicago sativa* CHS (AAA02824); Vv_CHS, *Vitis vinifera* CHS (CAA53583); Cs_CHS, *Cannabis sativa* CHS-like PKS (AAL92879); Hl_CHS1, *H. lupulus* CHS1 (CAC19808).

We characterized five PKS cDNAs, four from glandular hairs (*PKSG1*, *PKSG2*, *PKSG4,* and *PKSG5*) and one from seedlings (*PKSF3*). The last one was also identified in male and female flowers by RT-PCR and sequencing, while the expression of PKSG2 was also detected in leaves. Although a low expression of the known cannabis CHS-type PKS was reported in female flowers, glandular hairs, leaves, and roots (Raharjo *et al.*, 2004), we detected by RT-PCR that it is also expressed in male flowers. Southern blot analyses of *C. sativa* genomic DNA showed that at least four homologous *PKS* genes are present (Raharjo *et al.*, 2004). A phylogenetic analysis (Figure 3) of our cannabis PKSs revealed that they group together with other non-chalcone and non-stilbene forming enzymes and appear to be most closely related to the CHSs 2, 3, 4, and VPS from *H. lupulus*, while the known cannabis CHS-type PKS groups with chalcone-forming enzymes and is most closely related with *H. lupulus* CHS1, of which expression is highly specific in the lupulin glands during the cone maturation, but also it can be detected on all the plant (Matousek *et al.*, 2002a). *CHS2* has been detected from hop leaf and lupulin fractions and *CHS4* and *VPS* have been detected from the glandular tissue of hop cones, although no expression for *CHS3* has been found on any hop tissue (Okada and Ito, 2001; Novak *et al.*, 2003; Okada *et al.*, 2004). In regard to enzyme activities, CHS1 shows CHS activity with *p*-coumaroyl-CoA. VPS, CHS2, and CHS4 use isovaleryl-CoA and isobutyryl-CoA, but CHS2 and CHS4 reactions form byproducts. No reaction products have been detected for CHS3 enzyme activity (Okada *et al.*, 2004; Novak *et al.*, 2006). Probably, the right substrates have not been identified for this enzyme yet.

**Figure 4:**
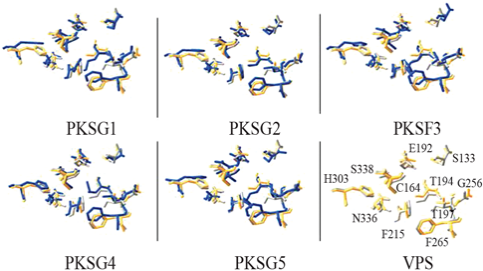
Relative orientation of the sidechains of the active site residues from the 3D model of *H. lupulus* VPS with the 3D models of *C. sativa* PKS. The corresponding sidechains in alfalfa CHS are shown in yellow and are numbered: for VPS in gray and for PKSs in blue.

A comparison of the 3D model of our PKSs, VPS, and alfalfa CHS2 predicted variations in the orientation of the active site residues which suggests a different substrate specificity regarding VPS (Figure 4).

The isolation and identification of PKSs with different enzymatic activity in one plant species has been reported, as well as the occurrence of PKS gene families in a species (Rolfs and Kindl, 1984; Zheng *et al.*, 2001; Samappito *et al.*, 2002; Matousek *et al.*, 2006). A number of points suggest the participation of several PKSs in the secondary metabolism of this plant. These are: the CHS- and STS-type, and olivetol-forming PKS activities from protein crude extracts from *C. sativa* (Flores-Sanchez and Verpoorte, 2008a), the expression and partial characterization of a PKS cDNA from leaves with CHS-type activities (Raharjo *et al.*, 2004), the characterization of four PKS cDNAs generated from mRNA of a glandular hair mixture and one from mRNA of seedlings, which is also expressed in female and male flowers (this study), and the small gene family of PKS detected in genomic DNA (Raharjo *et al.*, 2004). Recently, the crystallization of a cannabis PKS, called hexanoyl triacetic acid lactone (HTAL) or olivetol synthase (OLS), condensing malonyl-CoA and hexanoyl-CoA to form hexanoyl triacetic acid lactone or olivetol, was reported (Taguchi *et al.*, 2008; Marks *et al.*, 2009; Taura *et al.*, 2009). It has been proposed that pyrones or polyketide free acid intermediates undergo spontaneous cyclization to yield alkylresorcinolic acids or stilbenecarboxylic acids (Akiyama *et al.*, 1999); or that post-PKS modifying enzymes are required to form them (Austin and Noel, 2003; Eckermann *et al.*, 2003; Flores-Sanchez and Verpoorte, 2009). The homology of this protein with our PKSs was more than 97%. Although the differences in the amino acid residues from our PKSs and HTAL/OLS are small (Figure 1), probably because of the varieties of cannabis plant used, a complete biochemical characterization of the proteins encoded by *PKSG1*, *PKSG2*, *PKSF3*, *PKSG4*, and *PKSG5* is required to study and understand their function and diversity, as well as to learn more about signals or factors that could control their transcription and translation.

It is interesting that the cDNAs *PKSG1*, *PKSG2*, *PKSG4*, and *PKSG5* were generated from a combination of trichomes at different development stages. Under our greenhouse conditions, the beginning of the development of the trichomes on the perigonial bracts of female flowers was observed from 15 to 18 days after transferring the plants to a photoperiod regime. A full development of the glandular trichomes with presence of resin was observed from 30-35 days after initiation of the photoperiod regime. In addition, for the gland trichome isolation, flowers from two varieties of drug type (Skunk and Fourway) were used. Mahlberg *et al.* (1984) reported a glandular secretory system formed by three different forms of glandular trichomes on the epidermis of the outer surface of bracts from female flowers in *C. sativa*. The identification of non-glandular trichomes was also reported. A higher content of cannabinoids was detected in capitate-stalked glands than in capitate-sessile glands and appeared to be related to the gland age and type of cannabis plant. Bulbous glands are the smallest and there is no direct evidence for the presence of cannabinoids in them yet.

Olivetolic acid, an alkylresorcinolic acid, is the first precursor in the biosynthesis of pentyl-cannabinoids, and the identification of methyl- (Vree *et al.*, 1972), butyl- (Smith, 1997) and propyl-cannabinoids (Shoyama *et al.*, 1977) in cannabis plants suggests the biosynthesis of several alkylresorcinolic acids with different lengths of side-chain moiety (Figure S4). It is known that the activated fatty acid units (fatty acid-CoAs) act as direct precursors that form the side-chain moiety of alkylresorcinols (Suzuki *et al.*, 2003). Probably, more than one PKS-forming alkylresorcinolic acid or pyrone co-exist in cannabis plants. An analysis of cannabinoid content from our plant material showed the presence of THCA, a pentyl-cannabinoid, and THVA, a propyl-cannabinoid, in female flowers (Flores-Sanchez and Verpoorte, 2008a). As in *H. lupulus*, the presence of these PKSs could yield differences in the concentration of cannabinoids into becoming different varieties of *C. sativa*. Thus, the biochemical characterization of PKSG1, PKSG2, PKSG4, and PKSG5 will be carried out in the future in order to determine which PKS(s) is (are) involved in the pentyl-cannabinoid and/or propyl-cannabinoid pathways.

## Supplementary Material

The following online material is available for this article:

Table S1 Oligonucleotide primers and annealing temperatures used in this study.

Table S2 Homology percentage of *C. sativa* PKS ORFs with CHSs, STSs, and STCS.

Figure S1 Positions of degenerate primers and of the amplified PCR products, and sizes of PCR products, relative to *CHS*3 from *H. lupulus* (GenBank accession no. AB061022). Closed arrow heads indicate the sense and position of the degenerate primers relative to the amino acid sequences of the PKSs CHS, STS, and STCS. Amino acid numbering relative to *CHS*3 from *H. lupulus*.

Figure S2 Outline of RT-PCR and RACE for generation of PKS full-length cDNAs. Closed head arrows indicate the sense of the primers. The 5'- and 3'-ends were amplified from mRNA. PF, sense degenerate primer; PR, antisense degenerate primer. For nested amplification, gene-specific primers and amplification primers were used as nested primers.

Figure S3 Structural comparison of alfalfa CHS2 crystal structure with the 3D models from the deduced amino acid sequences of cannabis PKS cDNAs. The active site residues are shown as blue backbones; in alfalfa CHS structure naringenin and malonyl-CoA are shown as red and dark red backbones.

Figure S4 Proposed substrates for cannabis alkylresorcinolic acid-forming PKSs.

This material is available as part of the online article from http://www.scielo.br/gmb.

## Figures and Tables

**Table 1:** PKS full-length cDNAs generated from different *Cannabis sativa* tissues.

Tissue	Chemotype of plant	Variety	PKS cDNA	Full-length (bp)	5'-noncoding region (bp)	ORF (bp)	3'-noncoding region without the polyA tail (bp)
Glandular hairs	I or drug-type plant	Skunk and Fourway^*^	PKSG1	1455	98	1158	199
	I or drug-type plant	Skunk	PKSG2	1468	99	1158	211
	I or drug-type plant	Skunk and Fourway^*^	PKSG4	1472	108	1158	206
	I or drug-type plant	Skunk and Fourway*	PKSG5	1469	100	1158	211
Fiber female flowers	III or fiber-type plant	Kompolti	PKSF3	1503	94	1158	251
Male flowers	III or fiber-type plant	Kompolti	PKSF3	1466	97	1158	211
Seedlings	III or fiber-type plant	Kompolti	PKSF3	1434	105	1158	171

*A mixture of glandular trichomes from the Skunk and Fourway varieties in different stages of development and growth was used.

**Table 2:** Parameters of PKSs obtained from *Cannabis sativa* plant tissues.

Tissue	Name	ORF (aa)	Molecular mass (kDa)	pI
Glandular hairs	PKSG1	385	42.51	6.09
	PKSG2	385	42.61	6.09
	PKSG4	385	42.60	6.04
	PKSG5	385	42.57	5.98
Female and male flowers, and seedlings	PKSF3	385	42.57	6.09

aa, amino acids.
