# Supplementary material for: In silicio expression analysis of PKS genes isolated from Cannabis sativa L
Source: Genet Mol Biol. 2010 Dec 1;33(4):703–13. doi: 10.1590/S1415-47572010005000088 (PMC3036156; doi:10.1590/S1415-47572010005000088)
Supplement: Table S1 — Oligonucleotide primers and annealing temperatures used in this study. [file gmb-33-4-703-suppl1.pdf]

Supplementary Table 1. Oligonucleotide primers and annealing temperatures used in this study.

Primers	Sequence (5'→3')	Annealing temperature (°C)
Gene-specific primers		
1F	AATGGCTTGCTTGTTTCGTGGGCCTTCAGATTCTGACCTCGAATTACTAGTG	64
1R	CACTAGTAATTCGAGGTCAGAATCTGAAGGCCCACGAAACAAGCAAGCCATT	
2F	CATGACGGCTTGCTTGTTTCGTGGGCCTTCAGATTCTAACC	64
2R	GGTTAGAATCTGAAGGCCCACGAAACAAGCAAGCCGTCATG	
3F	CGAACCCGATGAGTCAGTTGGCGAAAGGCCGATATTTGAGTTA	63
3R	TAACTCAAATATCGGCCTTTCGCCAACTGACTCATCGGGTTCG	
4F	GTGGAGGAGAAGTTGGATCTGAAGAAGGAG	57
4R	CTCCTTCTTCAGATCCAACTTCTCCTCCAC	
5F	GTAGAGGAGAAGTTGCATCTGAAGAAGGAGAAGTTTGTGGAT	60
5R	ATCCACAAACTTCTCCTTCTTCAGATGCAACTTCTCCTCTAC	
Amplification primers		
PKSFw	ATGAATCATCTTCGTGCTGAGGGTCCGGCC	61
PKSRv	TTAATATTTGATGGGAACACTACGCACGACCAC	
PKSG2Rv	TTAATAATTGATCGGAACACTACGCAGGACCAC	62
PKSG5Rv	TTAATAATTGATGGGAACACTACGCAGGACCAC	62
Sequencing primers		
A (PKSG1)	CATGTTGGTAGTTGAGGTTCCAAAACTTGGGAAGGATGCTTGTGC	64
B (PKSG2)	GTCCCTCAGTGAAGCGTGTGATGATGTATCAACTAGGCTGTTA	63
C (PKSF3)	GCGCATCAACCACTGACATGCCCGGTGCAGACTACCATTGCG	68
D (PKSG4)	AATATGTGACAAAAGTATGATAAGGAAACGTAACTGTTTCTT	55
E (PKSG5)	GTGCAAAGGCCATCAAAGAATGGGGTCAACCCAAGTCTAAAAT	62
