# Supplementary material for: In silicio expression analysis of PKS genes isolated from Cannabis sativa L
Source: Genet Mol Biol. 2010 Dec 1;33(4):703–13. doi: 10.1590/S1415-47572010005000088 (PMC3036156; doi:10.1590/S1415-47572010005000088)
Supplement: Table S2 — Homology percentage of C. sativa PKS ORFs with CHSs, STSs, and STCS. [file gmb-33-4-703-suppl2.pdf]

Supplementary Table 2. Homology percentage of *C. sativa* PKS ORFs with CHSs, STSs and STCS.

PKS (species, ProteinBank accession numbers)	PKSG1	PKSG2	PKSF3	PKSG4	PKSG5
CHS-type PKS (*C. sativa*, **AAL92879**)	67	67	67	67	67
CHS 1 (*H. lupulus*,**CAC19808**)	66	66	66	66	66
CHS2 (*H. lupulus*, **BAB47195**)	69	68	69	69	69
CHS3 (*H. lupulus*, **BAB47196**)	**72**	**72**	**72**	**73**	**72**
CHS4 (*H. lupulus*, **CAD23044**)	**71**	**71**	**71**	**72**	**71**
VPS (*H. lupulus*, **BAA29039**)	**70**	**71**	**70**	**71**	**70**
CHS2 (Alfalfa, **AAA02824**)	65	65	65	65	65
2PS (*G. hybrida*, **P48391**)	61	61	61	60	61
STCS (*H. macrophylla*, **AAN76182**)	60	60	60	60	60
STCS (*M. polymorpha*, **AAW30010**)	52	53	52	53	53
STS (peanut, **BAA78617**)	60	60	60	60	60
STS (vine, **AAB19887**)	62	62	61	62	62
STS (*P. strobes*, **CAA87013**)	60	61	60	60	61
BBS (*P. sylvestris*, **CAA43165**)	59	60	59	59	60
BBS (*B. finlaysoniana*, **CAA10514**)	57	57	57	57	58
PCS (*A. arborescens*, **AAX35541**)	51	51	51	51	51
OKS (*A. arborescens*, **AAT48709**)	52	53	52	52	53
BPS (*H. perforatum*, **ABP49616**)	54	54	54	54	54
BIS (*S. aucuparia*, **ABB89212**)	55	55	55	55	56
HKS (*P. indica*, **BAF44539**)	55	55	55	55	56
ACS (*H. serrata*, **ABI94386**)	55	56	55	55	56
ALS (*R. palmatum*, **AAS87170**)	61	60	60	60	61
