# Supplementary material for: In silicio expression analysis of PKS genes isolated from Cannabis sativa L
Source: Genet Mol Biol. 2010 Dec 1;33(4):703–13. doi: 10.1590/S1415-47572010005000088 (PMC3036156; doi:10.1590/S1415-47572010005000088)
Supplement: Figure S1 — Positions of degenerate primers and of the amplified PCR products, and sizes of PCR products, relative to CHS3 from H. lupulus (GenBank accession no. AB061022). Closed arrow heads indicate the sense and position of the degenerate primers relative to the amino acid sequences of the PKSs CHS, STS, and STCS. Amino acid numbering relative to CHS3 from H. lupulus. [file gmb-33-4-703-suppl3.pdf]

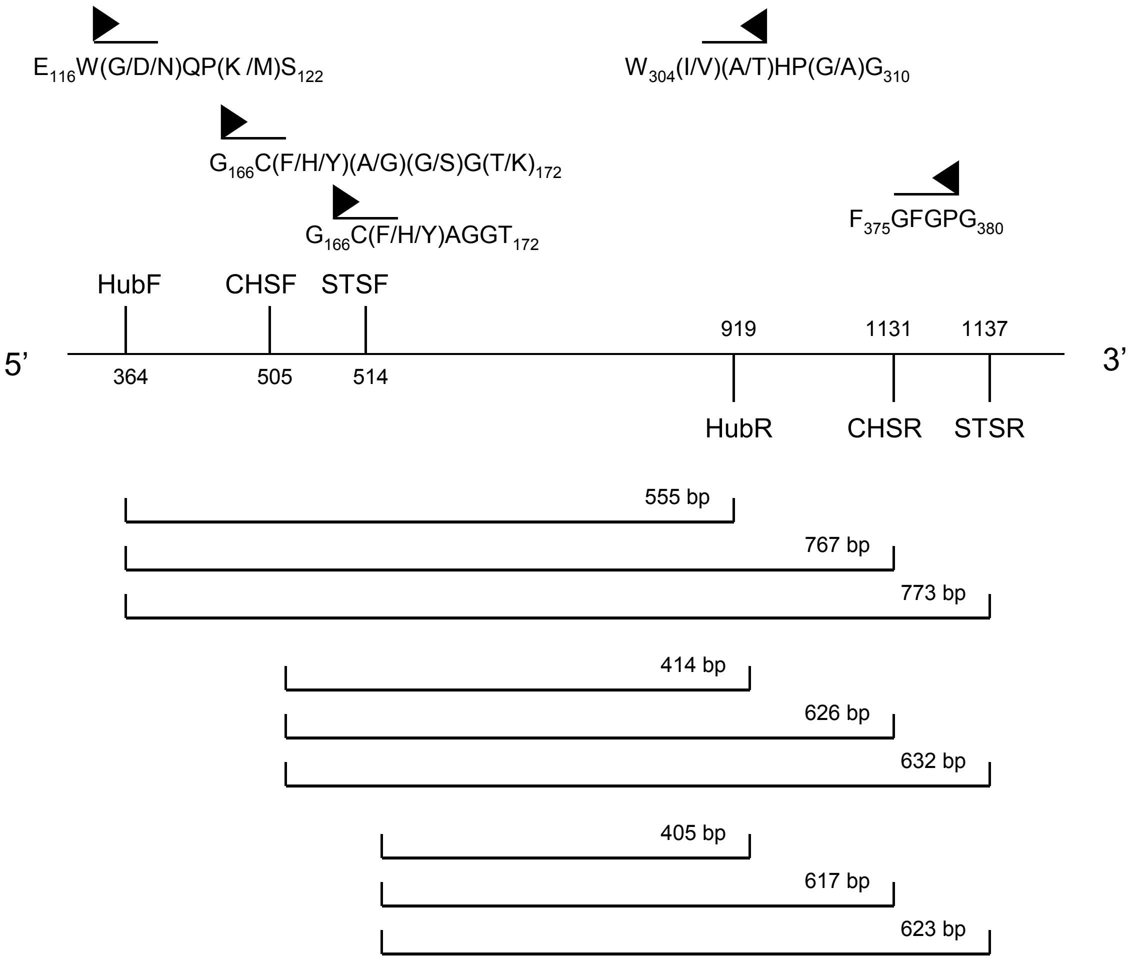

Supplementary Figure 1. Positions of degenerate primers and of the amplified PCR products, and size of PCR products, relative to *CHS*3 from *H. lupulus* (GenBank accession no. **AB061022**). Closed arrow heads indicate the sense and position of the degenerate primers relative to the amino acid sequences of the PKSs CHS, STS and STCS. Amino acid numbering relative to *CHS*3 from *H. lupulus*.
