# Supplementary material for: In silicio expression analysis of PKS genes isolated from Cannabis sativa L
Source: Genet Mol Biol. 2010 Dec 1;33(4):703–13. doi: 10.1590/S1415-47572010005000088 (PMC3036156; doi:10.1590/S1415-47572010005000088)
Supplement: Figure S2 — Outline of RT-PCR and RACE for generation of PKS full-length cDNAs. Closed head arrows indicate the sense of the primers. The 5'- and 3'-ends were amplified from mRNA. PF, sense degenerate primer; PR, antisense degenerate primer. For nested amplification, gene-specific primers and amplification primers were used as nested primers. [file gmb-33-4-703-suppl4.pdf]

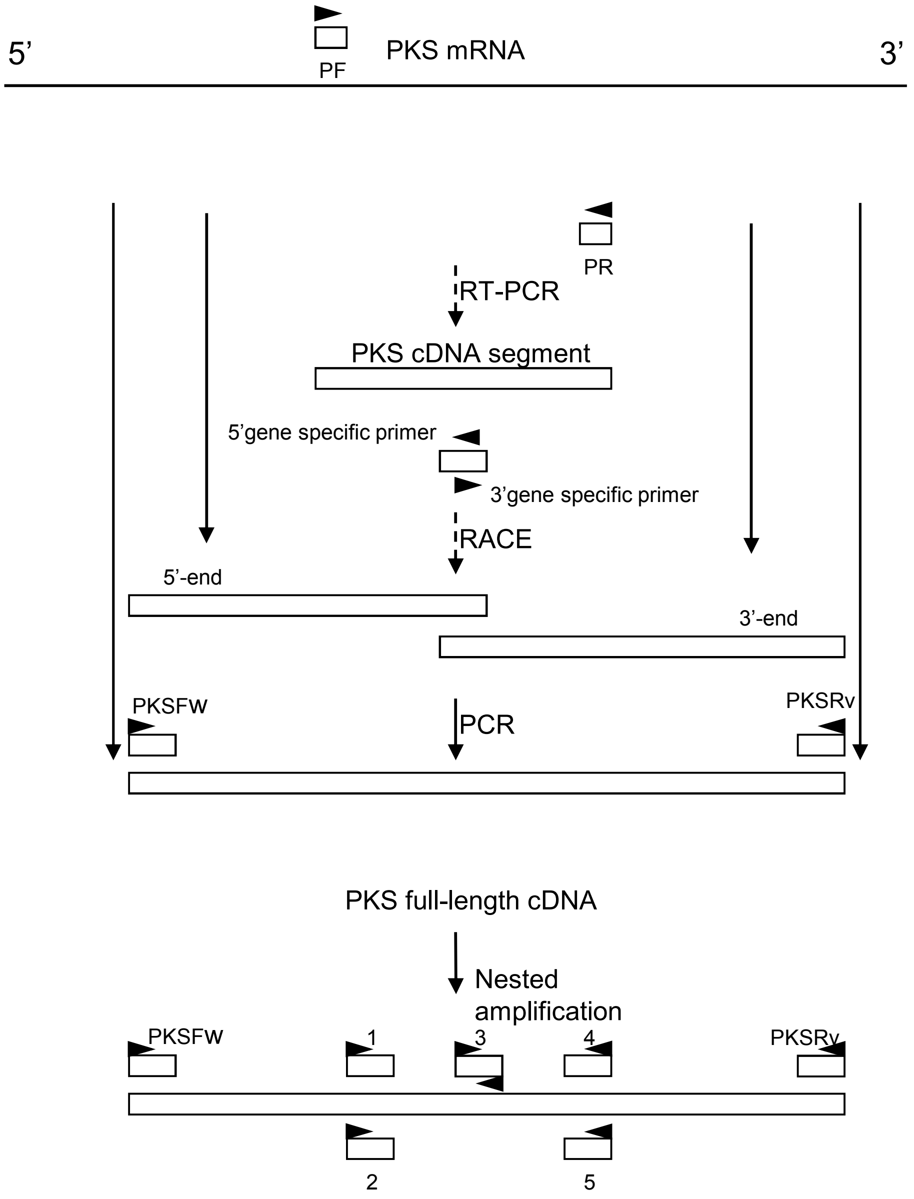

Supplementary Figure 2. Outline of RT-PCR and RACE for generation of PKS full-length cDNAs. Closed head arrows indicate the sense of the primers. The 5'- and 3'-ends were amplified from mRNA. PF, sense degenerate primer; PR, antisense degenerate primer. For nested amplification, gene-specific primers and amplification primers were used as nested primers.
