# Supplementary material for: In silicio expression analysis of PKS genes isolated from Cannabis sativa L
Source: Genet Mol Biol. 2010 Dec 1;33(4):703–13. doi: 10.1590/S1415-47572010005000088 (PMC3036156; doi:10.1590/S1415-47572010005000088)
Supplement: Figure S3 — Structural comparison of alfalfa CHS2 crystal structure with the 3D models from the deduced amino acid sequences of cannabis PKS cDNAs. The active site residues are shown as blue backbones; in alfalfa CHS structure naringenin and malonyl-CoA are shown as red and dark red backbones. [file gmb-33-4-703-suppl5.pdf]

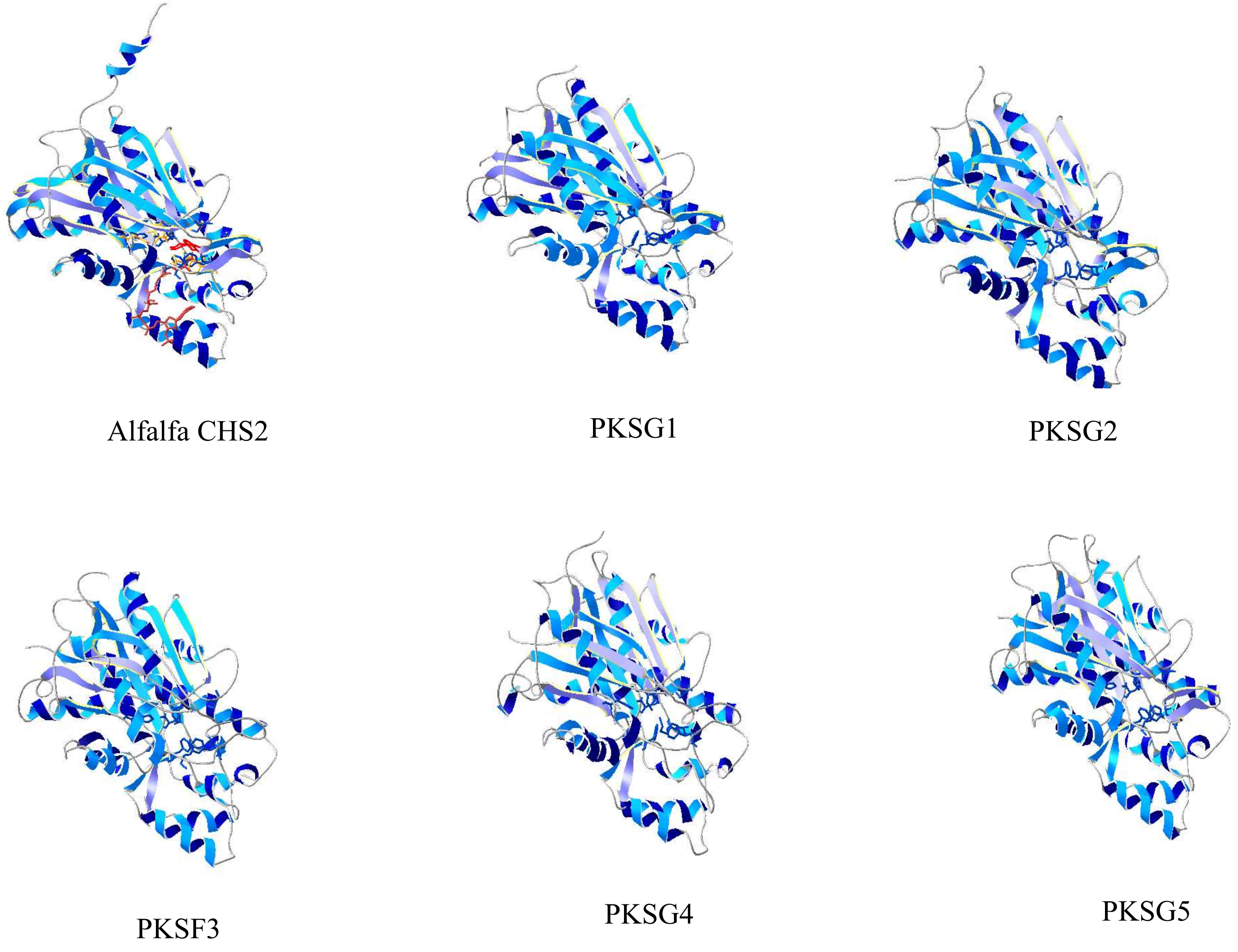

Supplementary Figure 3. Structural comparison of alfalfa CHS2 crystal structure with the 3D models from the deduced amino acid sequences of Cannabis PKS cDNAs. The active site residues are shown as blue backbones; in alfalfa CHS structure naringenin and malonyl-CoA are shown as red and dark red backbones.
