# Supplementary material for: In silicio expression analysis of PKS genes isolated from Cannabis sativa L
Source: Genet Mol Biol. 2010 Dec 1;33(4):703–13. doi: 10.1590/S1415-47572010005000088 (PMC3036156; doi:10.1590/S1415-47572010005000088)
Supplement: Figure S4 — Proposed substrates for cannabis alkylresorcinolic acid-forming PKSs. [file gmb-33-4-703-suppl6.pdf]

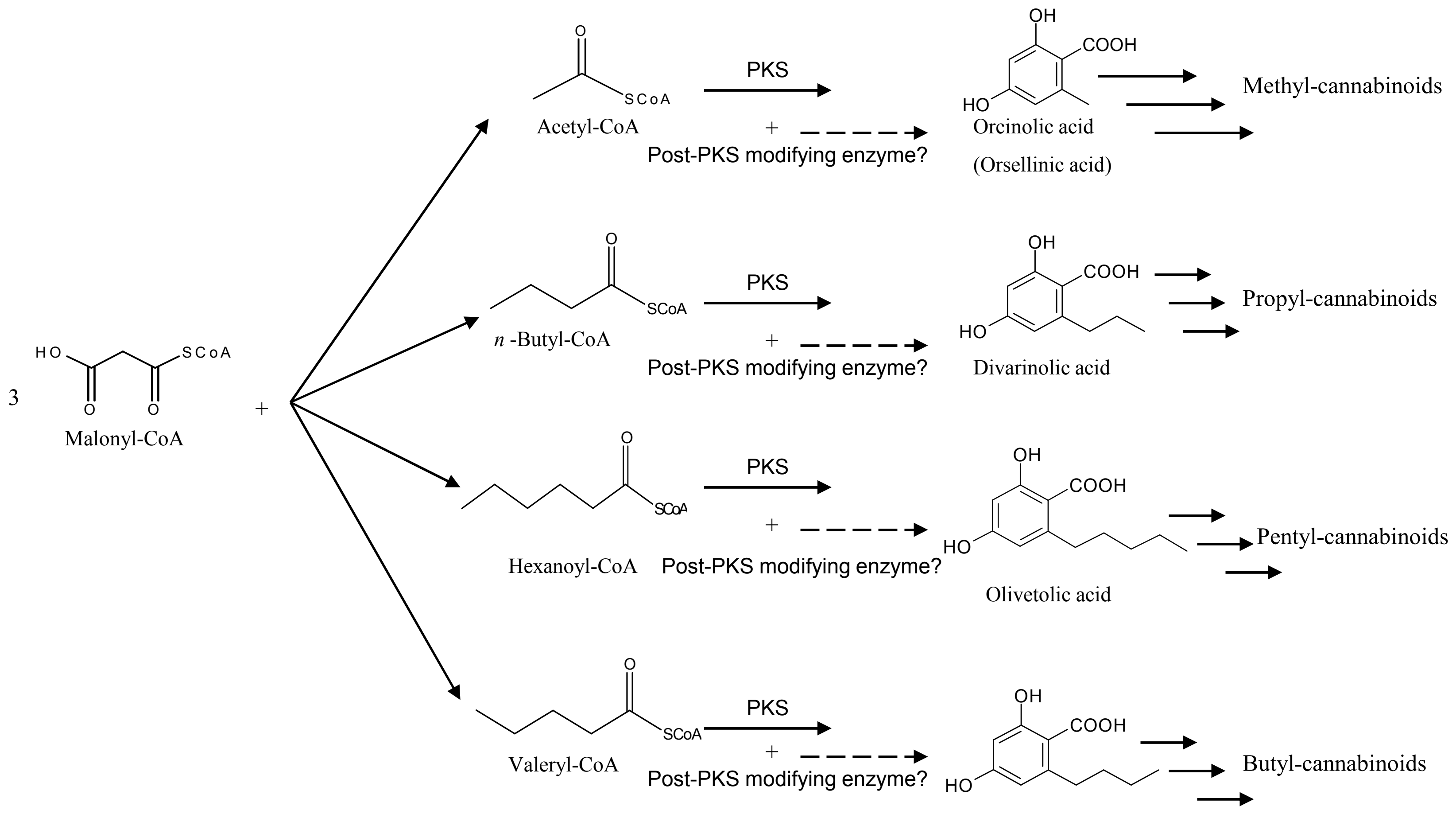


Supplementary Figure 4. Proposed substrates for cannabis alkylresorcinolic acid-forming PKSs
